# There is more to septic shock than arterial hypotension and elevated lactate levels: another appeal to rethink current resuscitation strategies!

**DOI:** 10.1186/s13613-018-0406-6

**Published:** 2018-04-27

**Authors:** Martin W. Dünser, Arnaldo Dubin

**Affiliations:** 1 Department of Anesthesiology and Intensive Care Medicine, Kepler University Hospital and Johannes Kepler University Linz, Linz, Austria; 2 Servicio de Terapia Intensiva, Sanatorio Otamendi y Miroli, Buenos Aires, Argentina

Sepsis is an umbrella syndrome created by clinicians to identify patients with an acute infection and a high risk of death. The Sepsis-3 definition group recognized new organ dysfunction as the key indicator determining whether a patient falls into the group with a high risk of death or not [1]. The same group has redefined septic shock as the combined presence of arterial hypotension [arbitrarily described by a mean arterial pressure (MAP) of 65 mmHg or less] and elevated lactate levels in the absence of hypovolemia [1]. Patients fulfilling these criteria were found to have a higher mortality compared to those presenting with sepsis alone [1, 2]. Despite of having an increased risk of death in common, patients with sepsis or septic shock represent a heterogeneous group of critically ill patients with different genotypes, comorbidities, underlying infections, clinical presentations (phenotypes), treatment requirements, and prognoses. Taking this into account, it is highly unlikely that standard treatment approaches, such as vasopressor therapy, are uniformly beneficial for all septic patients presenting with arterial hypotension and elevated lactate levels. So far, merely few studies have addressed this clinical and therapeutic heterogeneity of patients with sepsis or septic shock [3–5]. It is only the failure of (large) clinical trials to identify single therapies that improve survival [6] which reminds us about the fact that patients with sepsis share an increased risk of death but not the need for uniform treatment.

In a recent issue of the Annals of Intensive Care, Sacha et al. [7] reported the results of a study which highlights of our current misjudgment that single therapeutic approaches could benefit all patients with septic shock. The authors reviewed the prescription database of a tertiary care medical center in the USA for adult patients with septic shock (defined as a MAP < 65 mmHg) who received adjunct, fixed-dose arginine vasopressin (AVP) in addition to one or more catecholamine agents. They found that only 45% of their study patients achieved a MAP ≥ 65 mmHg and decreased their catecholamine requirements in response to AVP (AVP-responder). In the remaining 55% of the study population, initiation of AVP therapy was followed by a decrease in MAP values as well as an increase in catecholamine requirements and lactate levels. The only parameters the authors could identify from their database which differentiated between AVP responders and AVP non-responders were the location of admission (patients admitted to a non-medical intensive care unit were more likely to respond to AVP) and lactate levels before AVP initiation (the lower the lactate levels the more likely a patient responded to AVP). In comparison with AVP responders, AVP non-responders experienced a deterioration of organ function and higher mortality. Accordingly and together with many other factors, the hemodynamic response to AVP was found to be an independent predictor of death.

Due to the retrospective nature and the fact that the database contained only a limited set of variables, the authors could not report other hemodynamic parameters than MAP, catecholamine requirements and lactate levels. Given the striking difference in the response to AVP therapy, it is obvious that the two patient groups (AVP responders and AVP non-responders) relevantly differed in their hemodynamic status and hence their response to infusion of a strong vasoconstrictor drug such as AVP. This result underlines the common oversight of both intensivist and researchers that arterial hypotension and increased lactate levels in patients with sepsis would be signs of a uniform circulatory pathology. Basic physiology and recent research [8, 9] indicate that it is the delicate interplay of four key features determining cardiovascular failure and tissue hypoperfusion in septic shock: cardiac output, vascular resistance, microcirculatory function, and time. Based on this knowledge and clinical experience, one can assume that there were different groups of patients with different phenotypes of cardiovascular failure in the study population. Hypotensive patients with low lactate levels (AVP responders) may represent those subjects with a hyperdynamic circulation, a mild degree of microcirculatory dysfunction and a beneficial response to AVP therapy. These findings are in line with the results of a post hoc analysis of the large VASST trial reporting that only patients with normal lactate levels (< 1.4 mmol/L) at study enrollment who received AVP had a lower mortality than patients treated with norepinephrine alone. Interestingly, patients with lactate levels ≥ 4.4 mmol/L at randomization experienced a (nonsignificantly) higher mortality when treated with AVP compared to norepinephrine therapy [10]. Similarly, AVP non-responders in the study population of Sacha et al. [7] may well represent patients with either a hypodynamic circulation or late septic shock when the microcirculation had become uncoupled from the macrocirculation, meaning that increases in systemic blood flow do not translate into improved microcirculatory blood flow and tissue perfusion any longer.

Independent of these pathophysiologic speculations, the results of this study clearly underline that arterial hypotension alone is an inappropriate trigger for vasopressor or AVP therapy in critically ill patients (with sepsis). Instead of continuing our quest for a single therapeutic intervention miraculously capable of improving the mortality of a highly heterogeneous patient population, future clinical studies must elucidate the different and unique phenotypes hidden under the umbrella syndrome of sepsis and septic shock. Only with an improved understanding of the various pathophysiologic phenotypes presenting with arterial hypotension and elevated lactate levels in sepsis can adequate resuscitation strategies be developed and tested. Such resuscitation strategies must take these phenotypes with their specific time sensitivities to and indications for therapeutic interventions as well as appropriate resuscitation endpoints into account. In light of the missing answers to these fundamental questions of septic shock resuscitation, the current search for the ideal type of fluid, vasopressor, or inotropic agent appears of only minor relevance.

## Abbreviations

AVP: arginine vasopressin
MAP: mean arterial blood pressure
